# Geographic modeling and simulation systems for geographic research in the new era: Some thoughts on their development and construction

**DOI:** 10.1007/s11430-020-9759-0

**Published:** 2021-06-29

**Authors:** Min Chen, Guonian Lv, Chenghu Zhou, Hui Lin, Zaiyang Ma, Songshan Yue, Yongning Wen, Fengyuan Zhang, Jin Wang, Zhiyi Zhu, Kai Xu, Yuanqing He

**Affiliations:** 1 Key Laboratory of Virtual Geographic Environment (Ministry of Education of PRC), Nanjing Normal University, Nanjing, 210023 China; 2 State Key Laboratory of Resources and Environmental Information System, Institute of Geographical Sciences and Natural Resources Research, Chinese Academy of Sciences, Beijing, 100101 China; 3 School of Geography and Environment, Jiangxi Normal University, Nanchang, 330027 China; 4 Jiangsu Center for Collaborative Innovation in Geographical Information Resource Development and Application, Nanjing, 210023 China; 5 State Key Laboratory Cultivation Base of Geographical Environment Evolution (Jiangsu Province), Nanjing, 210023 China; 6 College of Oceanography and Space Informatics, China University of Petroleum, Qingdao, 266580 China

**Keywords:** Geographic characteristics, Development of geography, Regionality, Comprehensiveness, Complexity, Geographic modeling and simulation

## Abstract

Regionality, comprehensiveness, and complexity are regarded as the basic characteristics of geography. The exploration of their core connotations is an essential way to achieve breakthroughs in geography in the new era. This paper focuses on the important method in geographic research: Geographic modeling and simulation. First, we clarify the research requirements of the said three characteristics of geography and its potential to address geo-problems in the new era. Then, the supporting capabilities of the existing geographic modeling and simulation systems for geographic research are summarized from three perspectives: Model resources, modeling processes, and operational architecture. Finally, we discern avenues for future research of geographic modeling and simulation systems for the study of regional, comprehensive and complex characteristics of geography. Based on these analyses, we propose implementation architecture of geographic modeling and simulation systems and discuss the module composition and functional realization, which could provide theoretical and technical support for geographic modeling and simulation systems to better serve the development of geography in the new era.

## 1. Geographic characteristics and new requirements for problem solving

Regionality and comprehensiveness are generally viewed as two basic characteristics of geography and have guided the advancement and development of geographic research for a long time. Recently, with the deepening of geographic characteristic mining and geo-problem cognition, complexity has received more attention in geographic research and is now considered as the third characteristic of geography (Song et al., 2018, 2020b). However, regardless of whether the regionality, comprehensiveness or complexity is considered, the requirements that each characteristic poses in geographic research are not immutable. With the development of geographic research, the characteristics of geography have been analyzed on a deeper level beyond traditional geo-problem cognition, which puts forward several new requirements for related studies and problem-solving methods.

### 1.1 The regional characteristic of geography and its research requirements

As put by Prof. Bingwei Huang, geography aims to study the regional differences among complex bodies composed of different things in the world. Regionality has been viewed as the basic characteristic and the research core of geography (Zheng, 1998; Paasi, 2009; Fu, 2017). Regions are usually taken as geographic research units to analyze the interactions among natural and human elements, which can effectively reveal the genesis of geographic phenomena in regions, thereby promoting the coordinated development of man-land relationships (Zheng and Fu, 1999).

Regional geographic research is carried out from the regional perspective and chooses "differences" and "similarity" as the basic research points. Both concepts usually focus attention on differentiation and combination, or division and merging among regions, as well as the interconnection and evolution of geographic elements within regions (Li, 1982; Anselin, 1989; Zheng, 1998; Zheng et al., 2005; Lu, 2011). On the one hand, geographers employ data from observations or surveys and apply various methods, including classification and zoning, to explore the regularity of regional differentiation and describe geographic patterns (Tobler, 2004; Goodchild, 2004; Wu et al., 2016; Song et al., 2018; Zhu et al., 2018). On the other hand, in regional research, an array of methods, such as model construction and quantitative analysis, is used to explain the mechanisms of the interactions and dynamic processes of regional elements to understand various regional geographic processes (Gironás et al., 2010; Li et al., 2011; Li et al., 2018; Fan, 2018).

Recently, regional research has increasingly reflected the diversity of research problems and the relevance of research objects. Due to the differences in ranges, scales, and spatiotemporal locations of regional units, as well as the differences in the types, distribution patterns, interactions and evolutionary processes of geographic elements within regional units (Brown et al., 1996; Fu et al., 2001; Song et al., 2018), the geo-problems that need to be addressed through regional research are becoming increasingly diverse. They usually involve aspects that span an understanding of natural regions to the cognition of human society, and they also address different issues in various domains, such as hydrology, soil, climate, and biology, which require different types of work (Lin et al., 2006; Fu, 2018). In addition, traditional regional research usually focuses on the analysis of individual objects, which gives rise to an inadequate understanding of geo-problems in the new era. By contrast, regional geo-problem solving in the new era has increasingly focused on the associations and relationships among different research objects (Wu et al., 2016; Song et al., 2018). To deeply understand regional geo-problems, problems must be analyzed from not only multiple and wide perspectives but also from the entirety of perspectives. In addition, the mechanisms behind various phenomena and processes need to be fully considered, and the causal relationship of the generation and evolution of different regional geographic objects needs to be well understood. Moreover, geographers must pay attention to the integrated effects (e.g., soil-hydrological processes and ecological-economic processes) to explain the regional geographic phenomena and their evolutions (Seneviratne et al., 2010; Green et al., 2011; Cheng et al., 2014; Cheng and Li, 2015; Trinh et al., 2017).

### 1.2 The comprehensive characteristic of geography and its research requirements

As one of the traditional basic characteristics of geography, comprehensiveness is the overall property formed by the interactions of multiple natural and human elements in the geographic environment (Zonneveld, 1983; Waugh, 2000; Ni, 2003; Song et al., 2018, 2020b). Geographic phenomena and processes are generally the results of interactions among multiple geographic elements on the surface of the earth. These different geographic elements must be regarded as the research objects, and their relationships and driving processes should be analyzed to reveal the spatiotemporal patterns, interaction mechanisms and evolutionary laws of geographic environments (Chorley and Haggett, 2013; Wu et al., 2016; Lü et al., 2018; Fu, 2018).

Therefore, comprehensive geographic research usually takes geographic synthesis as the research object and focuses on various geographic elements to explore the relationships and interactions of elements behind geographic phenomena and processes (Fan, 2004; Fu, 2014). Regarding the implementation method, the development of comprehensive research can be divided into four stages: (1) Simple listing of natural and human elements, (2) spatial overlay of geographic elements based on geographic information systems (GIS), (3) comprehensive geographic analysis of different geographic elements, and (4) comprehensive understanding of geographic systems based on complexity theories (Song et al., 2018). The development of comprehensive research has promoted geographic research evolve from the single-element- and single-process-based spatial differentiation law and individual behavior research to multi-element- and multiprocess-based interrelationship and driving mechanism research, even extending to all-element and all-process systematic behavior and internal mechanism research on terrestrial surface systems (comprehensive research on man-land relationships) (Clarke, 2002; Ni, 2003; Xu et al., 2009; Chen F H et al., 2019; Song et al., 2020b).

Facing geographic environments with different structures and various changes, traditional comprehensive research struggles to holistically explain the reasons behind geographic phenomena. Thus, some new theories and methods need to be explored. Specifically, traditional comprehensive research influenced by "reductionism" theory generally analyzes and represents geographic phenomena and processes by disassembling and dissecting the geographic system and by listing and stacking geographic elements (Wen et al., 2006; Granell et al., 2013; Laniak et al., 2013; Song et al., 2018; Liu et al., 2019). However, whether from the perspective of data aggregation or model integration, the excessive division of research granularity might subjectively break the internal interactions among geographic elements, leading to the failure of the constructed digital geographic system to show the essence of the real geographic systems (Song et al., 2020b). Therefore, a higher-level abstract concept carrier than geographic elements must be discovered in the comprehensive research. The concept carrier needs not only to express the characteristics of geographic systems (e.g., their spatial structures, evolution processes, and interactions) but also to support the mining of geographic mechanisms and element relationships. Thus, an overall and comprehensive understanding of the geographic system can be represented in a "clear-context" and "clear-mechanism" manner (Lü et al., 2021). Furthermore, in terms of thinking modes, researchers need to consider both "holism" and "reductionism" and to regard the various geographic elements in the geographic synthesis as a coordinated whole at the conceptual level. Subsequently, the interactions among geographic elements could be fully understood (Fu, 2017), and the conceptual carriers of geographic phenomena and laws could be refined, constructed, and expressed. In terms of the research methods, data fusion, model integration and other methods must be used under the guidance of conceptual carriers. Thus, comprehensive geographic research with equal emphasis on "physical resources" and "conceptual knowledge" can be realized.

### 1.3 The complexity characteristic of geography and its research requirements

After the concepts of complexity science and complexity giant systems were proposed, discussions on the complexity of geography began worldwide (Qian et al., 1990; Cheng, 1999). With these concepts, geographers believe that the geographic system is also a complex system. Therefore, it is necessary to go beyond the traditional geographic methodology based on "reductionism" and explore more systematic research methods (Gan and Yang, 2004; O'Sullivan, 2004; Portugali, 2006; Li et al., 2010). Based on complexity theories, several geographic studies and applications have been carried out (Xu, 2013; Liu H M, 2014; Chen, 2015). In summarizing the geographic characteristics and challenges in the new era, Song et al. (2018) believe that complexity has become the third characteristic of geography.

According to the theory of geographic complexity, a geographic system is an open, complex and giant system with multi-element mixing, multiscale coupling, and multi-process interweaving (Shi et al., 2019; Song et al., 2020b). Compared with the comprehensive geographic research that takes geographic synthesis as the research object, complexity research focuses on geographic systems. This research was conducted on the characteristics of the geographic system, including its nonlinearity, burst (emergence), self-organization, self-similarity, and chaos (Song et al., 2018; Cheng et al., 2018), to fully understand the interactions among the internal and external geographic systems and reveal the spatiotemporal evolution mechanisms (Song et al., 2020a).

To address the complexity of the time, locations, interactions, and driving mechanisms of geographic systems, new requirements for the geographic research mode have been proposed. Specifically, due to the lack of personal knowledge and resources of geographers, it is often difficult to study the complexity of geography (Fan, 2004; Cutts et al., 2011; Lin et al., 2013a, 2013b; Lin and Chen, 2015; Chen and Lin, 2018). In order to solve complex geographic problems, the study of geographic complexity should be open to other researchers with different scientific backgrounds (Ni, 2003; Lü, 2011). Normally, active collaborations can better promote the integration of knowledge, resources, and skills (Voinov et al., 2016; Basco-Carrera et al., 2017). In addition, due to the complexity and uncertainty of geographic systems, the understanding of geographic systems usually changes and gradually becomes clear during the cycle of geographic research, which might result in tortuous research processes (Simão et al., 2009; Zare et al., 2020). Therefore, to address complex geo-problems, a fault-tolerant and explorable problem-solving process need to be considered and supported during complexity research.

## 2. Capability analysis of geographic modeling and simulation systems for geographic research

The geographic model is an important form to express geographic objects and geographic phenomena. Geographic modeling, as an important method of geographic research, is the process of abstracting and expressing geographic entities, geographic events, geographic processes, and geographic mechanisms. Geographic simulation is used for past inversions, future predictions, current simulations, and law discoveries , which is an important way to improve GIS geographic analysis ability. Traditionally, geographic models include data models (e.g., vector models, raster models, object-oriented models, and spatiotemporal data models) and analysis models (e.g., spatial analysis models, spatiotemporal statistical models, mechanism process models, and agent-based models) (Chen et al., 2018a). To bridge data models and analysis models, various computational grid models (e.g., structured grids, unstructured grids, hybrid grids, and nested grids) have been introduced (Chen et al., 2018b). Currently, various types of geographic models have been used in geographic research. For example, HASM (high accuracy surface modelling) has been proposed to serve regional geographic research. It can be employed for the surface modeling of geo-elements in different-scale regions and the expression of ecological environmental elements. By integrating the macro global information and the micro detailed information, HASM can be used to promote the comprehensive simulation and analysis of the earth's surface system. Moreover, the HASM method can be employed to explore related complexity problems (e.g., scale and error problems) in the simulation of the earth's surface system and its ecological environment elements based on the systematic theory and the fundamental theorem for eco-environmental surface modelling (Yue, 2011; Yue T X et al., 2016, 2020).

Focusing on the discovery of geographic mechanisms and laws, the analysis model is the main geographic model described in this paper, and the geographic modeling and simulation systems mainly refer to the system tools used for the construction and application of geographic analysis models in geographic research. With the development of geographic research, geographers have developed many geographic modeling and simulation systems, which differ in terms of model resources, modeling processes, and operational architectures. By summarizing the similarities and differences among geographic modeling and simulation systems from multiple perspectives, this paper analyzes the capabilities and deficiencies of these systems in coping with the geographic research, as shown in Figure 1.

### 2.1 Model resource analysis of geographic modeling and simulation systems

To address the diversity of geographic problems and the relevance of research objects, the model resources of geographic modeling and simulation systems are particularly important. In regional geographic research, model resources can directly affect the analysis capability of modeling and simulation systems for various geographic phenomena and processes in different regions. From the perspective of model resources, geographic models can be divided into individual models and integrated models (Oxley et al., 2004; Song and Leng, 2005; Lü, 2011). The individual model is an abstraction of geographic phenomena and processes expressed by formulas or rules. It can be used to summarize and describe the internal regularities and mechanisms of geographic systems (Badham et al., 2019). The integrated model is an integration of several geographic models (e.g., model components and model services). It can be used to express regional systems with multiple elements and processes (Elsawah et al., 2017; Chen M et al., 2019). Furthermore, the existing geographic modeling and simulation systems can be roughly divided into individual model systems for single-

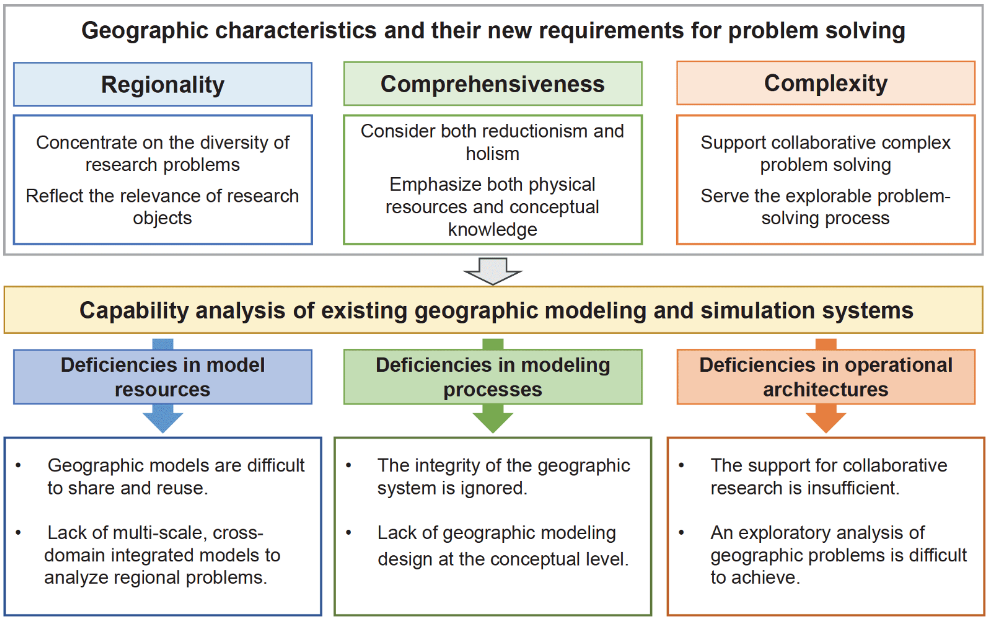

**Figure 1** Capability analysis of the existing geographic modeling and simulation systems in coping with geographic research.

domain geographic processes and integrated model systems for complex geographic processes (Chen et al., 2020).

To address various geographic problems from different regions, many single-domain process-oriented model systems have been developed, e.g., there are soil erosion prediction model systems (e.g., CSLE (Chinese Soil Loss Equation), WEPP (Water Erosion Prediction Project), EUROSEM (European Soil Erosion Model)) (Laflen et al., 1991; Morgan et al., 1998; Liu and Zhang, 2003), air quality prediction model systems (e.g., CMAx (Comprehensive Air Quality Model with Extensions), NAQPMS (Nested Air Quality Prediction Modeling System), CMAQ (Community Multiscale Air Quality Model)) (Wang et al., 2010; Huang et al., 2012; Su et al., 2012; Zhang et al., 2014a, 2015), hydrological process model systems for runoff analysis and numerical simulation (e.g., SWAT (Soil and Water Assessment Tool), MIKE SHE (MIKE System Hydrological European), FVCOM (Finite-Volume Coastal Ocean Model)) (Chen et al., 2003; Xu, 2010; Neitsch et al., 2011; Li X et al., 2018; Ramteke et al., 2020; Nixdorf et al., 2020), land use change simulation model systems (e.g., GeoSoS (Geographical Simulation and Optimization Systems), FLUS (Future Land Use Simulation Model)) (Li et al., 2011, 2017; Liu et al., 2017), and other single-domain process model systems (e.g., ADMS (Atmospheric Dispersion Management System), MASD (Multiple Adaptive Scenarios System Dynamic Model) and ANSWERS (Areal Nonpoint Source Watershed Environment Response Simulation)) (Singh et al., 2006; Liu D, 2014; Liu et al., 2018).

As an important tool for solving cross-domain and multi-process geographic problems, integrated model systems have attracted increasing attention. Thus, geographers and experts have focused on integrated geographic processes, such as the ecological-hydrological process in the Heihe River Basin (Cheng et al., 2014; Cheng and Li, 2015; Guo et al., 2018), the economic-social-environmental process in the Yangtze River Economic Belt (Li et al., 2019), and climate-hydrological processes in the region of the Shasta Dam (Trinh et al., 2017), to conduct various integrated model studies that consider multiple elements and processes in a region. Therefore, a series of integrated model systems have been developed for different geographic process simulations, such as the integrated simulation of global changes and terrestrial ecosystems (Tian et al., 2010), terrestrial water cycle process simulation (Tang et al., 2019), and simulation and evaluation of global carbon emission reduction programs (Wang et al., 2015).

Clearly, a large number of geographic modeling and simulation systems have emerged. Relying on model resources, these systems can support feature exploration and mechanism analysis for different geographic phenomena and processes. However, these geographic model resources supported by model systems are mainly oriented to a certain domain problem. Because of the multi-source and spatial distribution characteristics of these geographic model resources, and the differences in development language, operation environment, model structure and model data, it is often difficult to share and reuse these geographic model resources when solving various problems within the scope of the region (Lü, 2011; Lin et al., 2013a). Moreover, to address regional problems considering the relevance of research objects, the existing integrated model systems focus mainly on the interaction analysis of different elements in a certain region. When performing the relevance analysis of multi-scale and cross-domain geographic problems, the analysis and simulation capabilities of current model systems are still insufficient (Chen et al., 2020).

### 2.2 Modeling process analysis of geographic modeling and simulation systems

The geographic model is an abstract expression of geographic cognition, the interpretability and scientific rigor of which are determined by the geographic modeling process. On the one hand, abstract geographic cognition and its theories influence geographic model construction and guide the modeling process; on the other hand, the geographic modeling process supports the comprehensive geo-analysis process and cognition. From the perspective of the modeling process, geographers and experts first need to establish a conceptual understanding of the geographic system and then need to analyze the functional structure of the geographic model and the interaction among different functional modules to build a computable and executable geographic model system.

During the conceptual cognition and expression of geographic systems, the internal relationships and evolution laws of geographic phenomena and processes must be analyzed. In the early stages, geographers often made sketches to clarify geographic concepts. However, expressing geographic problems under a standardized format in this way was difficult (Renolen, 2000). Subsequently, to clarify and describe geographic problems, geographers began to use conceptual diagrams, for example, the conceptual framework used to represent spatiotemporal phenomena (Langran and Chrisman, 1988; Peuquet, 1994), the conceptual map model based on graphical representation (Wan et al., 2003), and the geographic conceptual modeling method for collaboration and sharing (Chen et al., 2009, 2011).

Based on the cognition and expression of geographic concepts, to analyze and describe the functional structure of geographic models, structure diagrams are applied to depict the logical structure of geographic modeling and simulation systems. For example, the Modular Modeling System and Spatial Modeling Environment use graphs to represent model variables and functions and connect different variables and functions in the diagram interface (Leavesley et al.,

1996; Costanza and Voinov, 2004); OpenFLUID represents various geographic entities as nodes by different shapes and colors and connects geographic entities through tree diagrams (Fabre et al., 2010); HIME (Heihe River Basin Integrated Modeling Environment) uses graphs to represent different functional modules in the model system, applies lines to describe the relationship between modules, and employs model structure diagrams to realize the construction of integrated models (Feng et al., 2008; Nan et al., 2011); and ArcGIS ModelBuilder uses different graphs and structural diagrams to describe the model data, behavior, and connections to support the construction of geographic models (Allen, 2011).

In summary, existing geographic modeling and simulation systems use mainly graphs and structure diagrams to describe and represent the different geographic elements and processes in the geographic modeling process. On this basis, the model variables, model data, functional components, and algorithm modules are linked to support the construction of geographic models. However, in terms of the thinking mode, the computer-designed node-link style is not in line with the cognitive habits of geographers. Moreover, model integration in the form of diagrams and connections is usually realized by disassembling the geographic elements and processes of geographic systems, which makes it difficult to accurately demonstrate a geographic phenomenon and its internal mechanism. Thus, the integrity of the geographic system may be neglected. In terms of the research method, the construction of an integrated model cannot be achieved by simply creating directed links among model components and data. To sort out the relationships among systems/subsystems, elements, and functions, especially to support integrated modeling for comprehensive scenarios, the entire modeling problem should be analyzed starting from the conceptual level. A set of geographic modeling and simulation solutions is needed to implement the process from visualized conceptual modeling to structured logical modeling and then to numerical computational modeling.

## 2.3 Operational architecture analysis of geographic modeling and simulation systems

Collaboration and exploration are important development trends of the research mode for complex geographic problems, and the development of the research mode imposes higher requirements on the operational architecture of geographic modeling and simulation systems. With the integration of information technologies and geographic theories, new changes have taken place in the storage, management and application of geographic simulation resources (Lin and Chen, 2015), and the operational architecture of the geographic modeling and simulation system has been continuously updated. Regarding the operational architecture, existing geographic modeling and simulation systems can be mainly divided into lumped systems, distributed systems, and service-based systems. These three architectures complement each other and support diversified geographic modeling and simulation research.

In geographic modeling and simulation systems with the lumped architecture, all simulation resources are centralized. Stand-alone calculation methods are typically used in these systems, such as the SIAT (Sustainability Impact Assessment Tool) (Verweij et al., 2010), the JGrass-NewAge system for hydrological forecasting and simulation (Formetta et al., 2014), and the SEAMLESS (System for Environmental and Agricultural Modeling; Linking European Science and Society) (van Ittersum et al., 2008; Janssen et al., 2011).

With the upgrading of operational architectures, distributed-architecture-based geographic modeling and simulation systems have emerged (Jing, 2014; You, 2017), such as FRAMES (Framework for Risk Analysis in Multimedia Environmental Systems) for ecosystem simulation (Whelan et al., 2014) and the Consortium of Universities for the Advancement of Hydrologic Sciences, Inc. (CUAHSI) Hydrologic Information System (HIS) hydrology project (Maidment, 2008). The simulation resources and running processes of these systems are distributed on multiple servers, which can support geographic modeling and simulation research in the distributed web environment (Tanenbaum and Steen, 2007). Moreover, some interfaces, frameworks and platforms used to promote distributed geographic modeling and simulation have received more attention, including OpenMI (Open Modeling Interface) (Moore and Tindall, 2005), ESMF (Earth System Modeling Framework) (Hill et al., 2004), DMIF (Distributed Model Integration Framework) (Belete et al., 2017), CSDMS (Community Surface Dynamics Modeling System) (Peckham et al., 2013), and OMS (Object Modeling System) (David et al., 2013).

With the development of Web services technology and SOA (Service Oriented Architecture), simulation resources are increasingly deployed and published as Web services in geographic modeling and simulation systems to support sharing and reuse (Wen et al., 2017; Chen et al., 2020; Niknejad et al., 2020), such as eHabitat, a web processing service for ecosystem simulation (Dubois et al., 2013), and AWARE, a water resource monitoring and prediction framework (Granell et al., 2010; Gan et al., 2020). In addition, based on the SOA, for the development of open science (Woelfle et al., 2011; Nosek et al., 2015), different geographers and experts have conducted preliminary explorations on open geographic modeling and simulation research modes, such as SWATShare (Rajib et al., 2016), HydroShare (Bandaragoda et al., 2019; Gan et al., 2020), and OpenGMS (Open Geographic Modeling and Simulation Systems) (Wen et al., 2013; Yue et al., 2015, 2018; Yue S S et al., 2016; Wang et al., 2018, 2020; Zhang et al., 2019, 2020; Chen M et al., 2019; Chen et al., 2020).

In general, the existing geographic modeling and simulation systems are based mainly on lumped and distributed operational architectures, which have been upgraded closely following the development of computer technologies. Moreover, these systems are developed mostly for specific domains. In terms of the research modes, most systems can support only the closed research conducted by specific institutions or groups. To meet the requirement of complex geographic problem solving, the closed modeling and simulation environment obviously cannot make full use of the existing simulation resources, which results in resource waste. More importantly, closed modeling and simulation systems struggle to support collaborative geographic research among multidomain geographers and experts. In addition, to address the complexity of geography, further exploration of the application scenarios and research modes of existing geographic modeling and simulation systems is necessary. With the development of open science, geographic research in the new era presents greater challenges to geographic simulation. Therefore, it is necessary to support collaborative exploratory geographic simulation and analysis with multiple experts, multiple hosts and multiple roles, and maintain the iterative process of solving complex problems. In this aspect, OpenGMS tries to form a virtual community for model resource reuse and integration in an open and cooperative way and thus provide an open geographic problem-solving platform that can be used transparently, efficiently and collaboratively (Chen et al., 2020).

## 3. Development directions of geographic modeling and simulation systems

Although the geographic modeling and simulation system is a typical software system, its main purpose is to serve the exploration and solution of geographic problems, and its essence is the construction and application of geographic analysis models. Therefore, the development of geographic modeling and simulation systems should not only comply with the development trend of information and software technologies but also focus on the basic characteristics of geography. For geographic modeling and simulation systems, the requirements of the existing systems should be analyzed, and the development direction to promote their continuous progress should be summarized, as shown in Figure 2.

### 3.1 Geographic model sharing to cope with the diversity of regional problems

To address various regional problems, geographic models must be fully utilized to analyze the distribution patterns and interactions of different geo-elements and the driving mechanisms of geo-processes. The emergence of new data and new technologies provides opportunities for accurately describing geographic environments at different spatio-temporal scales. Thus, the construction of new geographic models can occur continuously (Li and Li, 2014; Wang et al., 2014; Lu and Zhang, 2014; Dong et al., 2017). However, the isolated development of new models is costly. Existing geographic model resources must be fully and effectively used. Thus, apart from constructing new models, the sharing

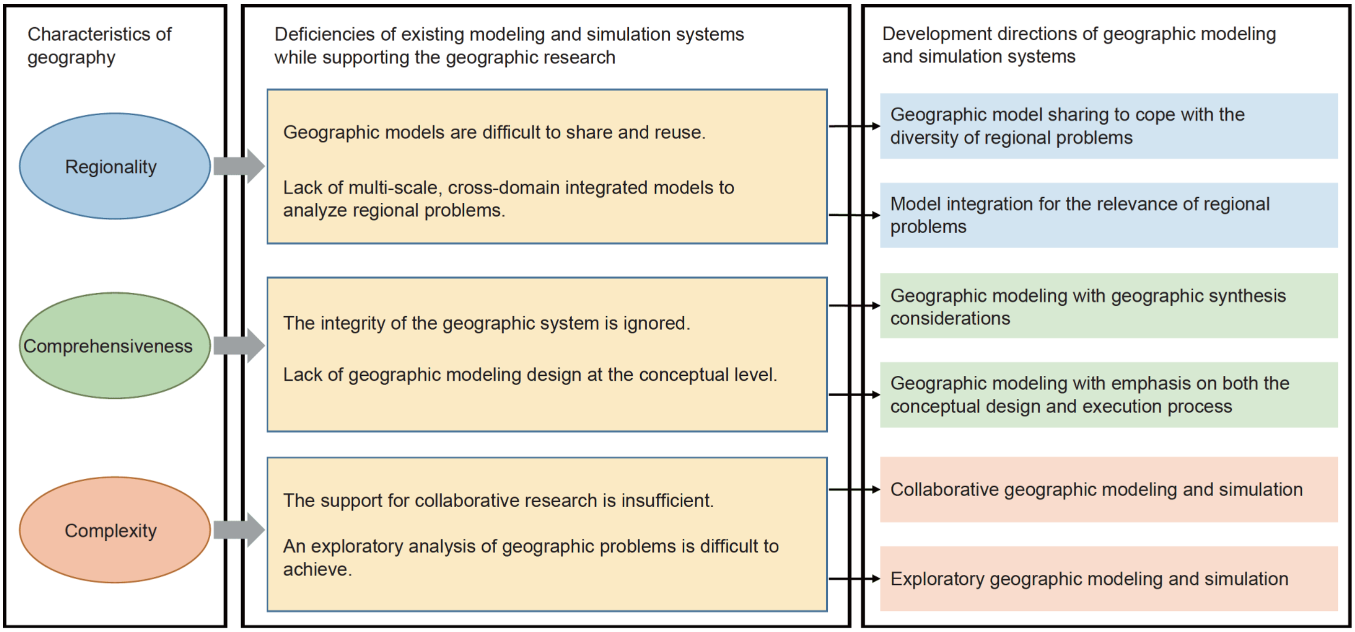

**Figure 2** Development directions of geographic modeling and simulation systems.

of geographic models should also be promoted. Because geographic model resources from different sources are usually heterogeneous, they are difficult to access and use to solve various geographic problems (Lü, 2011). To make full use of the existing models to solve regional problems, the heterogeneity and spatial dispersibility of geographic models must be overcome. Therefore, an important requirement for geographic modeling and simulation systems is to standardize multisource geographic models to shield against their heterogeneity (Hu, 2012; Müller et al., 2013; Harpham and Danovaro, 2015). These systems also need to have the ability to widely share models (Yue S S et al., 2016; Barton et al., 2020). On this basis, modelers could easily discover and access geographic models distributed in cyberspace to support the reuse of geographic models (Wen et al., 2013; Tan, 2018; Lü et al., 2019).

### 3.2 Model integration for the relevance of regional problems

Geographic model integration can be performed to integrate natural and human geographic elements at a certain scale, and it is an effective means to address the relevance of regional research objects (Laniak et al., 2013; Granell et al., 2013; Song et al., 2020a). Therefore, geographic modeling and simulation systems that analyze the relevance of regional issues need to support integrated modeling to discover the interaction between different research objects and explore the internal mechanism of the generation and evolution of each research object. Because of the work on single-domain process-oriented geographic modeling and the model sharing capability, a large number of model resources exist for solving geographic problems and sufficient model resources distributed in cyberspace are easy to reuse for integrated modeling. Hence, the integrated modeler also needs to select appropriate models to perform scale adaptation, computing grid conversion, data matching, and logical connecting of different models in accordance with the inherent geographic mechanism (Wen et al., 2006; Zhang et al., 2014a, 2014b; Li, 2016; Ma et al., 2019; Iwanaga et al., 2021). During the integration simulation process, the various submodels (or components and modules) participating in the integration must be controlled (Zhang et al., 2016; Zhang et al., 2018; Chen et al., 2020), and the error and uncertainty must be tracked and quantified to optimize the integrated modeling results (Yue T X et al., 2020). Therefore, geographic modeling and model systems need to vigorously develop an integrated modeling environment that can provide the corresponding strategies and tools to facilitate model selection, integration, and process control during the integrated modeling process to achieve not only multi-element and multiprocess perspectives but also full-element and full-process perspectives of integrated geographic modeling.

### 3.3 Geographic modeling with geographic synthesis considerations

In order to meet the overall research goal of integrated geographic problems, geographers must find an abstract carrier that can coordinate the cognition and expression of geography in the process of geographic modeling, so as to realize the interaction of spatial pattern, evolutionary process and geographic system. Geographic synthesis is an important way for geographers to explore comprehensive geographic cognition, such as comprehensive physical geographic regionalization and comprehensive division of regional environments (Fan, 2004; Liu et al., 2005). With the development of geographic research paradigms (e.g., empirical science, theoretical science, and system science), traditional methods, such as empirical cognition, element enumeration, and index weighting, often face the "easy to divide but difficult to synthesize" dilemma, and the modeling methods have attracted more attention for carrying out geographic synthesis (Song, 2016). Recently, in model studies, e.g., the Earth System Model, the Land Surface Process Model, the Global Ecosystem Model, and the Atmospheric/Ocean Model, on the one hand, geographers applied the results of geographic synthesis (e.g., climate zoning, geomorphological zoning, vegetation zoning, and land use zoning) and, on the other hand, promoted more comprehensive and refined geographic synthesis studies by using geographic modeling (Kawamura, 1994; Xia et al., 2009; Zhang et al., 2014). Notably, the methods combining statistical data analysis and mechanism process modeling (Dai and Han, 2014; Fan et al., 2017; Zhang, 2019) and the method of assimilating model results with observational data (Li et al., 2020) have gradually become important approaches advancing the research on geographic synthesis. Therefore, geographic modeling and simulation systems need to support a comprehensive understanding and expression of the inherent laws of geographic systems and break through the limitations of traditional geographic comprehensive research exemplified by "dividing first and synthesizing last" to achieve geographic modeling that considers geographic synthesis.

### 3.4 Geographic modeling with emphasis on both the conceptual design and execution process

In support of comprehensive geographic problem solving, geographic modeling needs to consider the conceptual design and implementation process, which is similar to the habit of thinking of geographers. Specifically, the entire modeling process needs to start from geographic concepts with which geographers are familiar, progress to the abstract analysis of internal logical relationships of the model, and culminate with the construction of computable geographic models. Thus, the whole modeling process, which contains visualized conceptual modeling, structured logical modeling, and numerical computational modeling, can be supported (Chen M et al., 2019; Chen et al., 2020). Conceptual modeling extracts various geographic elements from geo-entities and geo-systems, sorts out the interactions among elements, and constructs the conceptual expression of geo-systems (Chen et al., 2009, 2011). Logical modeling is usually based on the results of conceptual modeling and assumes responsibility for describing the internal structure and behavior of geographic models and establishing connections between geographic elements, geographic processes, model data, model parameters, and model components (Feng et al., 2008). Computational modeling builds the mapping from logical models to computational models by configuring related model resources, data resources, and computing resources and constructing the computable geographic model according to the prepared logical model. Therefore, geographic modeling and simulation systems need to support the whole process of geographic "conceptual-logical-computable" modeling, which can not only facilitate the conceptual analysis of interactions among geo-elements but also enable the use of data and models to conduct a practical exploration of geo-problem solutions.

### 3.5 Collaborative geographic modeling and simulation

To serve research on complex geographic systems, geographic modeling and simulation systems need to support collaborative work with different types of role engagement. In collaborative geographic modeling and simulation, participants with different roles can conduct different forms of geographic collaboration, including resource-based collaboration, knowledge-based collaboration, and interactive collaboration (Voinov et al., 2018; Bandaragoda et al., 2019; Elsawah et al., 2020). (1) In resource-based collaboration, different participants engage in geographic modeling and simulation by contributing their resources. By collecting resources from different participants (e.g., data resources, model resources, and computing resources), sufficient resources will be available to solve complex geographic problems (Basco-Carrera et al., 2017; Chen et al., 2020). (2) In knowledge-based collaboration, different participants conduct open geographic modeling and simulation by sharing related knowledge, including geographic knowledge and modeling methods. Knowledge sharing and negotiation among different participants can promote geographic modeling and simulation (Kelly et al., 2012; Almoradie et al., 2015). (3) In interactive collaboration, geographic modelers pay more attention to the core activities of geographic modeling and simulation, such as data processing (He et al., 2020) and model construction (Chen M et al., 2019). Through interactive collaboration, geographically distributed participants can directly perform modeling and simulation activities (Lü, 2011; Sun and Li, 2016). In addition, geographic modeling and simulation systems need to prepare strategies to support collaborative work. Thus, they can help to coordinate the specific work of geographically distributed geographers and experts and assist different participants in concentrating on their own tasks. All participants can bring their talents to bear in solving complex geographic problems (Ma et al., 2021).

### 3.6 Exploratory geographic modeling and simulation

It is often difficult to solve complex geographic problems through simple processes because knowledge and understanding of complex geographic systems is often inadequate. Therefore, geographic modeling and simulation systems should support not only open collaboration but also the solution exploration of geographic problems. First, to meet the needs of solving complex geo-problems, geographic modeling and simulation systems should be able to describe and archive the iterative exploration process that consists of different activities, including sensitivity analysis and uncertainty analysis (Jakeman et al., 2006; Badham et al., 2019; Hamilton et al., 2019; Koo et al., 2020a, 2020b; Razavi et al., 2021). In the modeling scenarios for different geographic problems, modeling and simulation systems should support participants in sharing their knowledge and experience and help to establish different modeling and simulation solutions that can lead different participants to high-level collaboration (Simão et al., 2009; Zare et al., 2020). Then, geographic modeling and simulation systems should have a unified evaluation system to support the evaluation of exploratory solutions. The engaged modelers can adjust and optimize the modeling and simulation solutions to improve their quality based on the evaluation results. Moreover, different modeling and simulation solutions are available for different problem exploration scenarios. Therefore, the different solutions must be compared and evaluated in order to choose a better one (Guo et al., 2012; Bennett et al., 2013; Yue et al., 2018; Yue S S et al., 2020). Finally, the geographic modeling and simulation systems need to support repeatable and reproducible work. This enables participants to effectively share and reuse existing modeling and simulation solutions (Munafò et al., 2017; Stark, 2018).

## 4. Design of implementation architecture for geographic modeling and simulation systems in the new era

Under the guidance of the development directions, geographic modeling and simulation systems need to design the system implementation architecture from three aspects:

sharing and reuse of the geographic simulation resources, collaborative geographic modeling, and open geographic simulation. Figure 3 shows the implementation architecture of the geographic modeling and simulation system for geographic research in the new era.

### 4.1 Service-based method studies on sharing and reusing geographic simulation resources

The development direction of geographic modeling and simulation systems for various regional geo-problems requires a system architecture design focused on service-oriented sharing and reuse of simulation resources. Geographic simulation resources (e.g., model resources, data resources, and computing resources) are the foundation of modeling and simulation. The service-oriented sharing and reuse methods mainly contain three parts: classification and description of geographic simulation resources, standardized encapsulation of geographic simulation resources, and service-based invocation of geographic simulation resources.

(1) To facilitate the management, sharing and reuse of heterogeneous geographic simulation resources, the characteristics of the simulation resources are selected as the starting point. On the basis of summarizing these characteristics, the classification, logical representation and attribute description of simulation resources can be developed to classify and describe the simulation resources structurally. The classification of simulation resources summarizes the resource categories according to different standards, such as the purpose of use (e.g., model, data, and calculation) and the domain of application (e.g., hydrology, soil, atmosphere, and ecology). Logical expression aims to describe geographic simulation resources in different ways (e.g., concept graphs, arithmetic logic, and invokable resources). Attribute description contains the description of attribute information (e.g., categories, introductions, and authors), operating behaviors (e.g., execution processes, inputs and outputs, and parameter configuration), environment dependencies (e.g., software environments and hardware environments) and other information.

(2) The standardized encapsulation of geographic simulation resources aims to encapsulate the simulation resources into web services to support the sharing and reuse of geographic simulations in an open web environment. First, simulation resource access is given to obtain multisource geographic simulation resources, such as componentized or

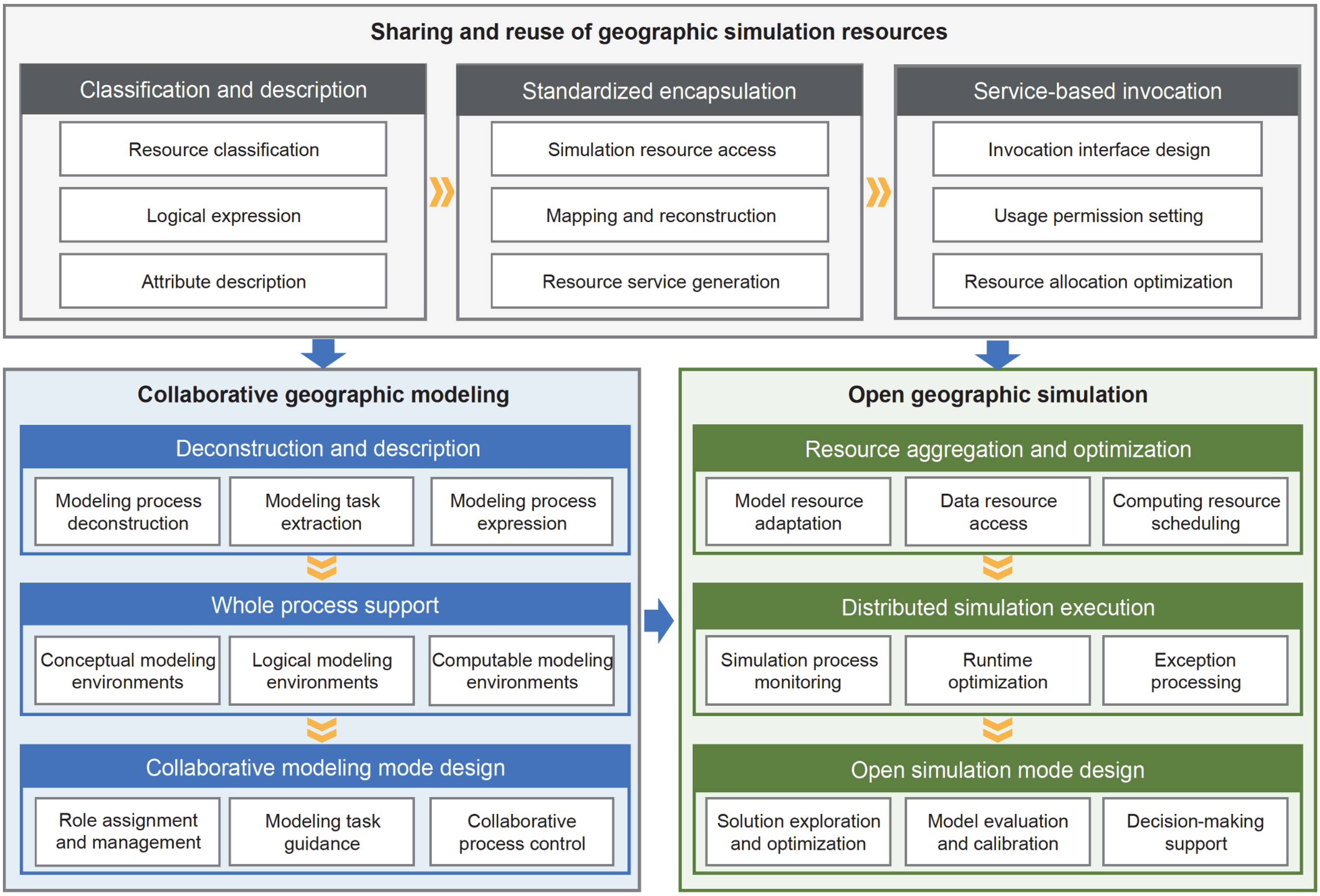

**Figure 3** Implementation architecture of a geographic modeling and simulation system.

service-based model resources, and data resources from cyberspace or local files. Then, based on the established standardized interfaces and the mapping rules between resource metadata and standardized interfaces, the mapping and reconstruction of heterogeneous simulation resources can be completed to support the service sharing of resources. Finally, based on the encapsulated resources, corresponding services of the simulation resources can be generated in the web environment for sharing and reuse.

(3) The service-based invocation of simulation resources is based on the geographic simulation service shared in the web environment. Geographic modeling and simulation systems prepare geographic model invocation interfaces to achieve geographic model reuse. Thus, several points need to be considered, including the resource invocation interface design, usage permission setting and resource allocation optimization. The geographic modeling and simulation system can realize different functions (e.g., model invocation and data refactoring) by designing geographic simulation resource invocation interfaces, thereby supporting the multidimensional reuse of simulation resources in the web environment. Moreover, different invocation interfaces are given corresponding usage permissions to ensure security and protect the copyright of simulation resources in the web environment. In addition, according to the usage status of simulation resources, the model and data can be migrated, and the computing nodes can be dynamically allocated. Therefore, the resources used for geographic simulation and calculations in the web environment can be scheduled, configured and optimized to improve reuse efficiency.

### 4.2 Collaborative method studies on geographic modeling

To solve comprehensive and complex geographic problems, collaborative comprehensive geographic modeling is required. The collaborative geographic modeling method can utilize various shared simulation resources and help provide model tools for geographic simulation. Specifically, it includes the deconstruction and description of the modeling process, the whole process support for geographic modeling, and the mode design of collaborative modeling.

(1) Geographic modeling for comprehensive geo-problems focuses mainly on two types of modeling: multimodule integrated modeling for addressing the relevance of regional problems and single-module model construction for geographic synthesis. First, the modeling process must be analyzed and deconstructed in accordance with the different geographic integrated modeling scenarios. Then, the core modeling tasks are extracted and the organization logics among different tasks are summarized, such as the algorithm selection and parameter calculation in the single-module model construction process, and the module selection and optimization in the model integration process. Finally, based on the abstract cognition of the types, goals, and contents of the core modeling tasks and their organization logics, the modeling process of different geographic models is expressed in a structured way. Thus, modelers from different backgrounds are guided into appropriate geographic modeling activities.

(2) To facilitate the geographic model construction that considers both the conceptual design and execution process, the collaborative geographic modeling method can provide environments for supporting the whole modeling process, including the conceptual modeling environment, the logical modeling environment, and the computable modeling environment. The premise of geographic synthesis is cognition of the geographic environment, and conceptual modeling is the basis for multiple experts to achieve common cognition. In the conceptual modeling environment, a series of tools and methods are provided to represent geographic objects and their interactions and constraints. Based on these tools and methods, construction from geographic phenomena and processes to geographic conceptual scenarios and to geographic conceptual models can be realized. In the logical modeling environment, modelers can use tools such as flowcharts, Unified Modeling Language (UML) diagrams, and workflows to clarify and describe the model logic, such as mathematical and rule expressions of a single-module model or the composition structure and data flow of a multimodule integrated model. In the computable modeling environment, single-module models can be quantified, for example, by model algorithm implementation and model parameter calculation, and scale-adapted, data-matched, and logically consistent geographic integration modeling can be achieved by selecting and matching the appropriate computing resources.

(3) Due to the complexity of geographic problems, geographers must support collaborative modeling among different participants during the entire geographic modeling process. Specifically, it is necessary to assign roles to different modelers according to their experience and background, including modeling experts, geographers, software engineers, and stakeholders. The permissions of different roles are managed and controlled during the modeling process. Then, to meet the different modeling goals and needs, participants with different roles are assigned and guided to engage in certain modeling tasks with which they are familiar. While performing modeling tasks, participants can negotiate with each other and conduct collaborative modeling activities. By designing a collaborative control mechanism, modeling managers can monitor the modeling process. By controlling multi-task conflicts and spatio-temporal inconsistencies, the quality of collaborative modeling in modeling activities can be maintained.

### 4.3 Method studies on open geographic simulation

To meet the requirement for solving complex geographic problems, geographic modeling and simulation systems should support open geographic simulation. Based on the architecture of open web environments, different geographers can conduct different activities, including collecting various simulation resources, exploring geographic simulation solutions, employing geographic models for geographic simulations, and evaluating, optimizing and applying simulation results. The key points of the open geographic simulation method include the aggregation and optimization of simulation resources, the distributed execution of simulation processes, and the mode design of open simulation.

(1) To carry out complex and changeable simulation tasks, various open simulation resources in the web environment must be converged and optimized. First, through model resource adaptation, shared model resources distributed in cyberspace can be effectively aggregated and applied for the simulation of geographic phenomena and processes. Moreover, data resources are the driving force behind the model execution and the bridge connecting different model modules. The data resource access strategy can enable various shared data resources to be acquired and used in geographic simulations; thus, it is the key to ensuring correctly conducted geographic simulations. In addition, computing resources, as the carrier of performing a simulation, play a fundamental supporting role in the entire simulation process. Therefore, by relying on the scheduling of computing resources, any idle shared computing resource in the web environment can be fully used to improve the efficiency of geographic simulation.

(2) In complex web and model execution environments, the status of the simulation resources that are scattered in the web environment is not always controllable. When the simulation scheme is running, the running status of the geographic models needs to be monitored and obtained in real time. Through simulation process monitoring, simulation modelers can optimize the model at runtime. Moreover, runtime exceptions must be captured in time and addressed accordingly so that the entire simulation process is always at the optimal status relative to the expected simulation target.

(3) The conducting of geographic simulation activities needs to be promoted in an open mode. First, the solving of complex geographic problems usually requires repeated explorations and attempts. After continuous optimizations and error corrections, more reasonable results can be obtained. Therefore, open exploration and optimization of geographic simulation solutions are required, and standardized exploration methods and optimization strategies for geographic simulations are needed. In an open simulation environment, to address unsatisfactory simulation results, different geographers and experts can discuss and analyze the causes and principles of the simulation results. If necessary, they can redesign the methods and evaluate and calibrate the models until the simulation purposes are achieved. Finally, in the open geographic simulation mode, the application of the geographic simulation results can also support open discussion and analysis, thereby providing assistance and support for geographic decision-making.

## 5. Conclusions

The development of system tools is driven by research needs, and it promotes the improvement of research capabilities. The requirement for geographic research has led to the innovation of scientific research methods and tools in geography and has brought new challenges to related software systems. As one of the main tools of geographic research, geographic modeling and simulation systems have received wide attention. However, how to evaluate the adaptability and effectiveness of geographic modeling and simulation systems has not yet been confirmed. Starting from the geographic characteristics—regionality, comprehensiveness and complexity—this paper analyzes the fundamental needs of geographic research in the new era to promote the design of the geographic modeling and simulation system architecture. The designed geographic modeling and simulation system architecture will also promote related geographic research and provide important technical support for the development of geography in the new era, thereby laying a basic foundation for the construction of a new generation of geographic simulators.

**Acknowledgements** *We appreciate the detailed suggestions and comments from the responsible editor and anonymous reviewers. We express heartfelt thanks to members of the OpenGMS team. This work was supported by the National Natural Science Foundation of China (Grant Nos. 41930648, 41622108 & U1811464).*

**Open Access** This article is licensed under a Creative Commons Attribution 4.0 International License, which permits use, sharing, adaptation, distribution and reproduction in any medium or format, as long as you give appropriate credit to the original author(s) and the source, provide a link to the Creative Commons licence, and indicate if changes were made. The images or other third party material in this article are included in the article's Creative Commons licence, unless indicated otherwise in a credit line to the material. If material is not included in the article's Creative Commons licence and your intended use is not permitted by statutory regulation or exceeds the permitted use, you will need to obtain permission directly from the copyright holder. To view a copy of this licence, visit http://creativecommons.org/licenses/by/4.0/.
